# Lack of an Association between Angiotensin Receptor Blocker Based Therapy and Increased Risk of Cancer: Evidence from Large Observational Studies

**DOI:** 10.1371/journal.pone.0119775

**Published:** 2015-03-19

**Authors:** Yuan Yang, Fan Zhang, Laura Skrip, Han Lei, Suxin Luo, Kai Lu, Dayi Hu

**Affiliations:** 1 Department of Cardiovascular Medicine, the First Affiliated Hospital of Chongqing Medical University, Chongqing, China; 2 School of Public Health and Health Management, Chongqing Medical University, Chongqing, China; 3 Department of Epidemiology of Microbial Diseases, Yale School of Public Health, New Haven, Connecticut, United States of America; Universität Bochum, GERMANY

## Abstract

**Background:**

A previous meta-analysis of randomized controlled studies that were not designed to investigate cancer as a primary outcome suggested that ARB-based therapy is associated with increased risk of cancer; however, results of recent observational studies considering the association have been contradictory. This study sought to evaluate the association between angiotensin receptor blocker (ARB)-based therapy and risk of cancer by conducting a meta-analysis of observational studies.

**Methods:**

Relevant articles published before February 2014 were identified by searching PubMed and the Cochrane Library. Pooled relative risks (RRs) were determined using a random effects model and were used to assess the strength of association between use of ARB-based therapy and risk of cancer.

**Results:**

Six retrospective cohort studies involving a total of 3,827,109 participants and four case-control studies involving a total of 193,029 cases were included. The present study found that ARB-based therapy was not significantly associated with an increased risk of cancer (RR = 0.87, 95%CI: [0.75, 1.01]). However, an analysis including only cohort studies suggested a significantly decreased risk of cancer among individuals with any history of ARB use as compared to those with no history of ARB use (RR = 0.80, 95%CI: [0.55, 0.95]); no significant association was found between ARB use and risk of cancer when the case-control studies were separately considered (RR = 1.03, 95%CI: [0.93, 1.13]). Subgroup analyses showed that use of ARB-based therapy was associated with decreased risk of lung cancer (RR = 0.81, 95%CI: [0.69, 0.94]); however, no significant associations were found with the other cancer sites investigated. Furthermore, no association was observed upon adjustment by type of ARB drug. No publication bias was detected.

**Conclusion:**

Overall, ARB-based therapy was not associated with increased risk of cancer. However, its use may be related to decreased incidence of lung cancer; this finding should be considered carefully and confirmed with further studies.

## Introduction

Angiotensin receptor blockers (ARBs) serve as first-line treatment for patients with hypertension. The potential relationship between ARB use and risk of cancer has been studied widely, although associations between increased risk and administration of ARBs as monotherapy have been modest or non-significant [1, 2]. A 2010 meta-analysis of eight randomized controlled trials (RCTs) provided evidence that ARB-based therapy was associated with slightly, yet significantly increased incidence of cancer (relative risk (RR): 1.08; 95% confidence interval (CI): [1.01, 1.15]) [1]. However, a subsequent meta-analysis of 70 RCTs found no association between ARBs as monotherapy and increased risk of cancer [2].

ARBs act on the renin-angiotensin-aldosterone system. Angiotensin II is the main mediator in the renin-angiotensin system (RAS), which is generated by the activation of angiotensin I through the angiotensin converting enzyme. However, angiotensin II is not only an effective hypertensive agent, but also is related to cell growth [3–9]. Expression of RAS mediators has therefore been demonstrated in cancer tissues [10]. There are several potential mechanisms for the involvement of ARBs in carcinogenesis at specific sites. For instance, in vitro, telmisartan has been shown to inhibit human urological cancer cell growth through early apoptosis by peroxisome proliferator-activated receptor (PPAR)-γ [11], which provides a strong link between lipid metabolism and the regulation of gene transcription [12]. In hormone-refractory prostate cancer cells, ARBs have been observed to inhibit angiogenesis by transcriptional factor Ets-1 which regulates angiotensin II-mediated vascular pathophysiology [3] and genes involved in endothelial function and angiogenesis [4]; ARBs have likewise been shown to inhibit angiogenesis by hypoxia inducible factor-1 alpha (HIF-1a) which plays a role in vascular endothelial growth factor (VEGF) induction by angiotensin II in vascular smooth muscle cells (VSMC) [5, 6]. Furthermore, local angiotensin II generation has been demonstrated in human gastric cancer, with tumor progression facilitated through the activation of ERK1/2 and NF-kappa B [7]. For lung cancer, Batra et al [13] found that angiotensin II elevated cytosolic free calcium in human lung adenocarcinoma cells via activation of AT1 receptors. Lastly, Gallagher [14] suggested that Ang-(1–7) inhibited the lung cancer cell growth through the activation of an angiotensin peptide receptor and may represent a novel chemotherapeutic and chemopreventive treatment for lung cancer.

Since the publication of both meta-analyses and laboratory researches results, large observational studies investigating the potential association between ARB use and risk of cancer have been widely conducted [15–24]. Many of these studies have methodologically extended beyond the RCTs included in the 2010 meta-analyses in that they use cancer as the primary outcome and they considered risk for specific cancer sites [16, 17, 20, 24]. In response to this recent accumulation of evidence, we sought to evaluate the association between ARB-based therapy and risk of cancer by conducting a meta-analysis of large cohort and case-control studies.

## Methods

### Search strategy

Relevant studies were identified through PubMed and the Cochrane Library databases by using the following search terms: 1) Cancer: “cancer” OR “carcinoma” OR “malignancy” OR “neoplasm” OR “tumor”; 2) ARB drugs: “angiotensin-receptor blocker” OR “ARB” OR “losartan” OR “valsartan” OR “candesartan” OR “irbesartan” OR “eprosartan” OR “telmisartan” OR “olmesartan”; and 3) Study design: “cohort studies” OR “follow-up studies” OR “prospective studies” OR “cross-sectional studies” OR “longitudinal studies” OR “retrospective studies” OR “case control studies”. Additionally, the reference lists of articles retrieved from databases, conference abstracts, and the publication lists of experts on ARB therapy were reviewed for other possible studies. This search strategy was conducted according to the PRISMA (Preferred Reporting Items for Systematic Reviews and Meta-Analyses) Statement [25] and all studies ultimately included in the analysis had been published on or before Feb 25, 2014.

### Selection criteria

Based on a review of the retrieved articles’ titles/abstracts and full texts, studies were chosen if they met all of the following criteria: (i) the study was designed as a prospective or retrospective cohort study or as a case control study; (ii) the primary exposure investigated was patients’ use of ARB-based therapy, and the primary outcome was the incidence of cancer; (iii) the articles provided hazard rate (HR) or relative risk (RR) estimates and the corresponding 95% confidence intervals (CIs), the size of the baseline samples, the total number of cases, the total number of years of follow up, and confounders for adjustment; (iv) when multiple publications reported on the same study, the most recent article was selected; (v) the publication language was limited to English and Chinese; (iv) the randomized controlled trials were excluded.

### Data extraction

The following information was extracted independently by two authors (Yang Y and Zhang F) from each article: first author, year of publication, study location, subject ethnicity, study period, duration of follow-up, source population, mean sample age at baseline, number of cases, assessment of ARB-based therapy use, ascertainment of cases, adjustment for covariates, hazard ratio (HR) or relative risk (RR), and the corresponding 95% confidence interval.

### Assessment of Study Quality

Study quality was scored using the Newcastle-Ottawa-Scale (NOS). The NOS was developed for quality assessment of non-randomized observational studies such as those included in the current meta-analysis. Details on the NOS “star system” can be found elsewhere [26]. Scoring for quality assessment was independently conducted by two authors (Yang Y, Zhang F); their results were compared and a third party (Hu D) intervened if consensus could not be reached.

### Statistical analyses

Relative risk (RR) estimates were used to measure the association between ARB-based therapy and risk of cancer. The hazard ratio was considered as a RR for the present analysis. One study [18] reported hazard ratios separately for colon and rectal cancers; using the method reported by Hamling [27], we combined the results for these two sites for subgroup analysis of colorectal cancer.

Inter-study heterogeneity was assessed using the Cochran’s *χ* ^2^-based Q test and the I-squared test [28]. If no significant heterogeneity (defined as *P*>0.10 or I^2^ <50%) was found, the pooled RR estimate was determined with the fixed effects model (Mantel-Haenszel); the random effects model (DerSimonian and Laird) was used in the case of significant heterogeneity [29]. Stratification analyses by study design, ethnicity, cancer site, and type of ARB drug were conducted as a way of addressing inter-study heterogeneity. Further more, meta-regression was also conducted when necessary.

To consider particular study elements that could impact the overall findings, the sensitivity analyses were conducted by excluding studies with the following characteristics. Accordingly, we considered the change of results upon removal of (i) studies that had follow up of less than four years; (ii) studies with less than 2000 cases; (iii) studies with NOS scores less than 7; (iv) studies adjusted with insufficient confounders (less than 3 confounders) [24], or no adjustments [20]; and (v) studies that had users of angiotensin converting enzyme inhibitors (ACEIs) [15, 22], diuretics, and/or beta-blockers [19] as their comparison group. Furthermore, to determine whether any study had particularly strong influence over the findings, such as due to assessed study quality, a leave-one-out approach was also engaged.

Furthermore, possible publication bias was considered using Egger’s linear regression test [30] and the Begg’s rank correlation test [31]. All statistical tests were conducted using STATA software (Version 11). A P-value less than 0.05 for any test or model was considered to be statistically significant unless otherwise indicated.

## Results

### Literature search

**Fig 1:**
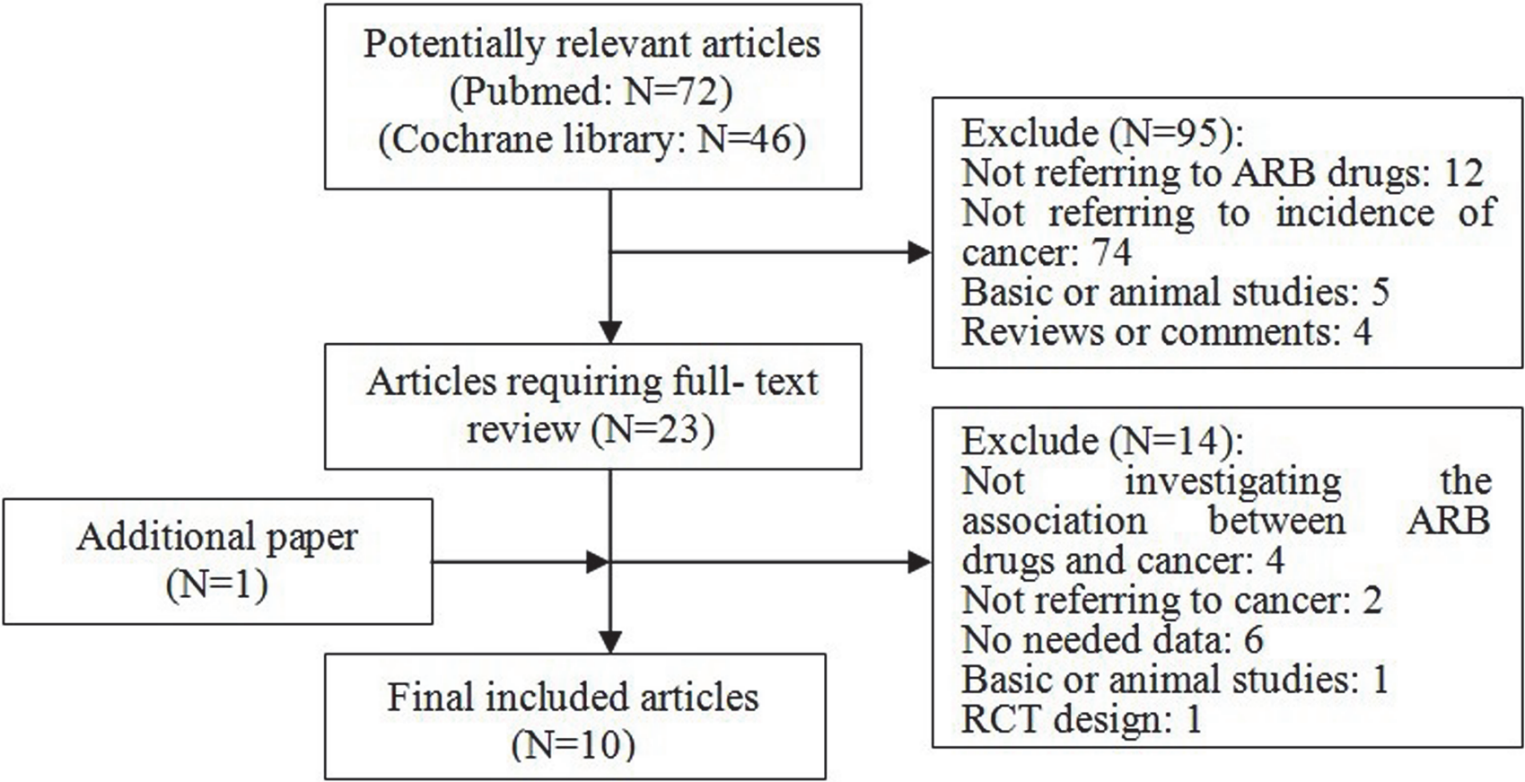
Flow Chart of Study Selection. Flow chart shows literature search for relevant studies about the association between ARB-based therapy and risk of cancer.

Initially, 118 citations were identified. Ninety five publications were excluded after review of titles and abstracts, mainly because they did not treat cancer as the primary outcome. Full-text review was conducted for 23 studies, of which four [32–35] were excluded from the analysis because they did not refer to the association between ARB-based therapy and cancer diagnosis. Six more articles [36–41] were excluded since they did not provide all information to be extracted. Two more articles [42, 43] were excluded because they did not refer to the incidence of cancer. Moreover, one randomized controlled trial [44] and one animal study [11] were excluded. After review of the references of the remaining studies, one additional study [15] was found to meet the inclusion criteria and was added. A total of 10 studies [15–24], all published in English, were ultimately included. (**Fig. 1**)

### Eligible studies

The included 10 studies were published between 2009 and 2013. Three studies took place in Taiwan of China, two in the United States, two in the United Kingdom, two in Denmark, and one in the Netherlands. Seven studies enrolled only Caucasian participants and participants of the remaining three studies were of Asian background. Six studies were designed as retrospective cohort studies and four were case-control studies. At baseline, in the cohort studies, a total of 3,827,109 participants were included and 59,530 cases were detected during the follow-up period. In the case-control studies, 193,029 cases and 1,019,459 controls participated. The length of follow-up ranged from 2.9 to 7.8 years (one study [17] did not provide data on follow-up period). The ARB drugs used by participants included losartan, irbesartan, valsartan, eprosartan, candesartan, olmesartan, and telmisartan. Most of the studies confirmed disease status according to ICD-9, medical record review, or pathology reports. The exposure was status as an ever or current user of ARB treatment, and the comparison group was made up of non-ARB users [16–18, 20, 21, 23, 24], angiotensin converting enzyme inhibitor (ACEI) users [12, 22], or users of diuretics and/or beta-blockers [19]. All 10 studies included adjustment for more than three variables, such as age, sex, body mass index (BMI), socioeconomic status, diagnosis of diabetes, concomitant use of medications including ACEIs, beta blockers (BB), calcium channel blockers (CCB), diuretics, or statins. The details are shown in **Table 1**.

**Table 1:** Characteristics of ten included studies of ARB and risk of cancer.

Study	Location/Period	Follow-up (years)	Comparison	Type of ARB	Number of sample		Sites of cancer*	Adjustments	Study conclusion
Cohort studies					Total of sample	Number of patients			
Rao GA, 2013 [16]	USA/1999–2010	4.5	Never using ARB	Losartan, irbesartan, valsartan, candesartan, olmesartan, telmisartan	1228960	6923	Lung	Age, race, smoking, BMI, DM	HR: 0.74, 95% CI: [0.67, 0.83]
Rao GA, 2013 [17]	USA/2003–2009	NA	Never using ARB	Losartan, irbesartan, valsartan, candesartan, olmesartan, telmisartan	543824	8775	Prostate	Age, race, smoking, BMI, DM	HR: 0.91, 95% CI: [0.84, 0.99]
Wang KL, 2013 [18]	Taiwan of China/1997–2009	4.8	Never using ARB	Valsartan, losartan, irbesartan, telmisartan, candesartan, olmesartan	85842	4394	All	Concomitant medication including ACEI, BB, CCB, diuretics, statin, and tumor markers.	The cumulative incidence of cancer was 4% (ARB users) and 6% (ARB nonusers) (HR: 0.58, 95% CI [0.55, 0.62])
Pasternak B, 2011 [22]	Denmark/1998–2006	2.9	ACEI user	Losartan,eprosartan, valsartan, irbesartan, candesartan, telmisartan, olmesartan	317158	10168	All	Calendar year, age in 5-year intervals, sex, socioeconomic class, degree of urbanization, number of hospitalizations in the previous 3 years, Charlson comorbidity index, and use of other antihypertensives (BB, thiazides, CCB)	RR: 0.99, 95% CI: [0.95, 1.03]
Huang CC, 2011 [23]	Taiwan of China/1995–2002	5.7	Never using ARB	Losartan, valsartan, irbesartan, candesartan, telmisartan	109002	9067	All	Age, gender, DM, coronary artery disease, hyperlipidemia, heart failure, valvular heart disease, ischemic stroke, chronic renal disease, and concomitant antihypertensive agents	HR: 0.66, 95% CI: [0.63, 0.68]
Bhaskaran K, 2012 [15]	UK/1995–2010	4.6	ACEI user	Losartan, eprosartan, valsartan, irbesartan, candesartan, telmisartan, olmesartan	1542323	20203	All	Age, sex, BMI, smoking, alcohol, DM, hypertension, heart failure, statin use, index of multiple deprivation score, calendar year	HR: 1.03, 95% CI: [0.99, 1.06]
Case-control studies					Number of control	Number of case			
Azoulay L, 2012 [19]	UK/1995–2008	6.4	Diuretics and/or beta-blockers	Losartan, eprosartan, valsartan, irbesartan, candesartan, telmisartan, olmesartan	410167	41059	All	Excessive alcohol use, BMI, smoking, diabetes, previous cancer, and ever of aspirin, statins, and NSAIDs	RR: 1.00, 95% CI: [0.96, 1.03]
Hallas J, 2012 [20]	Denmark/2000–2005	7.8	Never using ARB	Losartan, eprosartan, valsartan, irbesartan, candesartan, telmisartan, olmesartan	597668	149417	All	NA	RR: 1.12, 95% CI: [1.06, 1.18]
Chang CH, 2011 [21]	Taiwan of China/2000	7.4	Never using ARB	Losartan, valsartan, irbesartan, candesartan, telmisartan	5104	1281	All	Antihypertensive agents, fast-acting human insulins, chronic liver disease, biguanides, nephropathy, glinides, retinopathy, cardiovascular disease, statins, and socioeconomic status	RR: 0.94, 95% CI: [0.80, 1.10]
Koomen ER, 2009 [24]	Netherlands/1991–2004	3.0	Never using ARB	NA	6520	1272	Melanoma	The total number of unique medical diagnoses and the use of statins	RR: 1.00, 95% CI: [0.70, 1.50]

ARB: angiotensin receptor blocker; ACEI: angiotensin-converting enzyme inhibitor; BB: beta-blocker; BMI: body mass index; CCB: calcium channel blocker; HR: hazard ratio; RR: rate ratio; NA: not available; NSAIDs: Non-steroidal anti-inflammatory drugs; UK, United Kingdom; USA: United States of America; DM: diabetes mellitus.

*All includes breast, colon, liver, lung, rectum, stomach, prostate and haematological.

After quality assessment using the NOS, two studies [20, 24] received six stars; three studies [16–18] received seven stars; three studies [19, 22, 23] received eight stars; and two studies [15, 21] received nine stars (**Table 2**).

**Table 2:** Newcastle-Ottawa-Scale scores for the quality assessment of including studies.

Studies	Selection	Comparability	Outcome	Total stars
**Cohort studies**				
Rao GA, 2013 [16]	★★★	★★	★★	**7**
Rao GA, 2013 [17]	★★★	★★	★★	**7**
Wang KL, 2013 [18]	★★★★	★	★★	**7**
Pasternak B, 2011 [22]	★★★★	★★	★★	**8**
Huang CC, 2011 [23]	★★★★	★★	★★	**8**
Bhaskaran K, 2012 [15]	★★★★	★★	★★★	**9**
**Case-control studies**				
Azoulay L, 2012 [19]	★★★★	★★	★★	**8**
Hallas J, 2012 [20]	★★★★		★★	**6**
Chang CH, 2011 [21]	★★★★	★★	★★★	**9**
Koomen ER, 2009 [24]	★★★	★	★★	**6**

### ARB-based therapy and risk of cancer

#### Overall analysis

**Fig 2:**
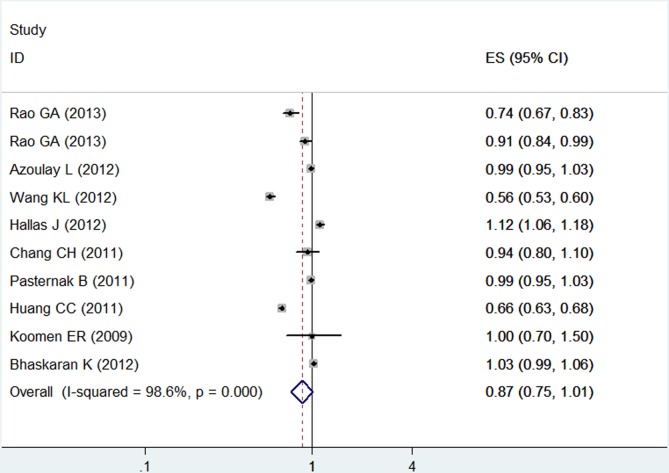
Forest plot of ARB-based therapy and risk of cancer. Forest plot shows association between ARB-based therapy and risk of cancer. CI = confidence interval; ES = estimates.

Based on the combined results of the 10 cohort studies, compared with non-ARB users or ACEI users, the patients reporting ever or current use of ARB-based therapy did not experience significantly increased risk of cancer (RR = 0.87, 95%CI: [0.75, 1.01], P = 0.07) under the random effects model (Heterogeneity: I^2^ = 98.6%). (**Fig. 2**)

Using the results of the six cohort studies, it was shown that ARB-based therapy was associated with decreased risk of cancer (RR = 0.80, 95%CI: [0.55, 0.95], P = 0.03, I^2^ = 99.1%). However, upon analysis of the results from the four case-control studies, no significant association was found (RR = 1.03, 95%CI: [0.93, 1.13], P = 0.60, I^2^ = 79.1%). (**Table 3**)

**Table 3:** Subgroup analyses of the included studies.

	No. of studies	OR(95%CI)	P	I^2^	Publication bias				Effect model
					Z value	P_Begg_	t value	P_Egger_	
Studies design									
Cohort	6	**0.80 [0.55, 0.95]**	**0.03**	99.1%	0.38	0.71	−0.67	0.54	Random
Case-control	4	1.03 [0.93, 1.13]	0.60	79.1%	0.34	0.73	−0.08	0.94	Random
Ethnicity									
Asian	3	**0.69 [0.57, 0.83]**	**<0.001**	95.3%	0.00	1.00	0.41	0.75	Random
Caucasian	7	0.97 [0.91, 1.04]	0.36	89.2%	0.75	0.45	−1.10	0.32	Random
Site of cancer									
Breast cancer	7	0.88 [0.76, 1.02]	0.09	85.3%	1.80	0.07	−2.45	0.06	Random
Lung cancer	8	**0.81 [0.69, 0.94]**	**0.01**	90.2%	0.87	0.39	−0.81	0.45	Random
Prostate cancer	6	1.01 [0.89, 1.12]	0.68	82.2%	0.38	0.71	0.11	0.91	Random
Colorectal cancer	5	0.94 [0.81, 1.09]	0.41	74.0%	−0.24	1.00	−0.85	0.46	Random
Type of ARB									
Losartan	4	0.90 [0.74, 1.09]	0.90	86.7%	0.34	0.73	1.43	0.29	Random
Candesartan	4	0.83 [0.68, 1.01]	0.06	80.1%	−0.34	1.00	7.42	0.02	Random
Irbesartan	4	0.89 [0.65, 1.12]	0.40	89.8%	0.34	0.73	7.58	0.02	Random
Valsartan	4	0.83 [0.58, 1.18]	0.30	86.2%	1.02	0.31	1.23	0.34	Random
Telmisartan	4	0.91 [0.59, 1.41]	0.78	86.0%	0.34	0.73	2.42	0.14	Random

ARB: angiotensin receptor blocker; OR: odds ratios

#### Subgroup analysis

A subgroup analysis was conducted according to participant race. For studies conducted with Asian samples, the meta-analysis revealed a significantly decreased risk of cancer among ever or current ARB users as compared to non-ARB users or ACEI users (RR = 0.69, 95%CI: [0.57, 0.83], P<0.001, I^2^ = 95.3%). In contrast, no significant associations were found in Caucasian populations (RR = 0.97, 95%CI: [0.91, 1.04], P = 0.36, I^2^ = 89.2%). Subgroup analyses according to cancer site suggested a significant association between ARB-based therapy and decreased risk of lung cancer (RR = 0.81, 95%CI: [0.69, 0.94], P = 0.01, I^2^ = 90.2%). However, no significant association was found in the analyses with other sites of cancer (breast: RR = 0.88, 95%CI: [0.76, 1.02], P = 0.09, I^2^ = 85.3%; prostate: RR = 1.01, 95%CI: [0.89, 1.12], P = 0.68, I^2^ = 82.2%; colorectal: RR = 0.94, 95%CI: [0.81, 1.09], P = 0.41, I^2^ = 74.0%). Similarly, no association between risk of cancer and ARB use was observed upon evaluating the results of studies grouped according to ARB drug type (losartan: RR = 0.90, 95%CI: [0.74, 1.09], P = 0.29, I^2^ = 86.7%; valsartan: RR = 0.83, 95%CI: [0.68, 1.01], P = 0.06, I^2^ = 80.1%; irbesartan: RR = 0.89, 95%CI: [0.65, 1.12], P = 0.40, I^2^ = 89.8%; candesartan: RR = 0.83, 95%CI: [0.58, 1.18], P = 0.30, I^2^ = 86.2%; telmisartan: RR = 0.91, 95%CI: [0.59, 1.41], P = 0.78, I^2^ = 86.0%). Furthermore, when we separately evaluated the studies with follow-up of at least five years, no statistically significant relationship was observed between ARB-based therapy and incidence of cancer (RR = 1.01, 95%CI: [0.95, 1.07], P = 0.80, I^2^ = 99.1%). (**Table 3**)

### Meta-regression analysis

We performed meta-regression analysis to assess whether the incidence of cancer was related to ethnicity. The result showed that the incidence of cancer in ARBs users was associated with ethnicity (r = 0.37, P = 0.006, 95%CI: [−0.60, −0.14], with adjusted R-squared = 46.52%).

### Sensitivity analysis

Sensitivity analyses were performed to consider the influence of various exclusion criteria and to identify potential sources of heterogeneity in the association between use of ARB-based therapy and risk of cancer. Exclusion of studies with less than four years of follow up produced a pooled RR of 0.84 (95%CI: [0.69, 1.02], P = 0.08, I^2^ = 99.0%). A sensitivity analysis excluding studies with NOS scores of six stars or less led to a pooled RR of 0.83 (95%CI: [0.71, 0.99], P = 0.03, I^2^ = 98.8%). Removing studies with under 2000 cases produced a pooled RR of 0.85 (95%CI: [0.72, 1.01], P = 0.06, I^2^ = 98.9%), and restricting the analysis to studies including non-ARB users as the comparison group yielded a pooled RR of 0.82 (95%CI: [0.66, 1.02], P = 0.08, I^2^ = 98.4%). Finally, the pooled RR was 0.83 (95%CI: [0.71, 0.99], P = 0.03, I^2^ = 98.8%) when we removed the studies with insufficient confounders [20, 24].

Furthermore, the leave one out procedure did not alter our conclusions with overall combined RR subsequently ranging from 0.85 (95% CI: [0.72, 0.99], P = 0.04, I^2^ = 98.6%) to 0.91 (95% CI: [0.80, 1.05], P = 0.21, I^2^ = 98.1%).

### Publication bias

**Fig 3:**
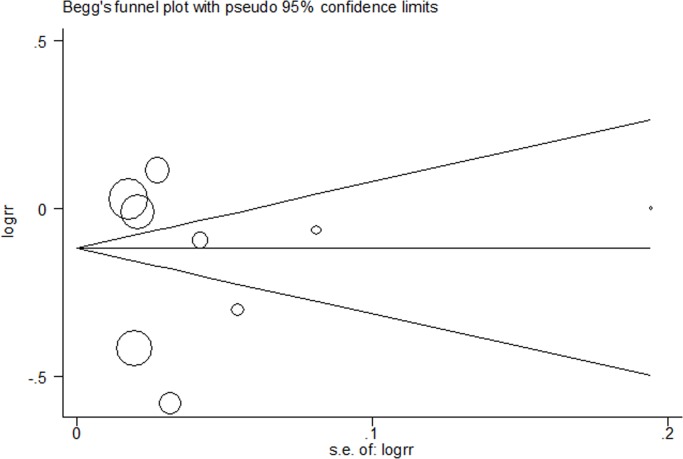
Funnel plot of ARB-based therapy and risk of cancer. Funnel plot is relatively symmetrical which suggests there is no significant publication bias.

A funnel plot (**Fig. 3)** was produced to determine whether significant publication bias was present. The relatively symmetrical shape of the plot was interpreted as a lack of significant publication bias. The results of the Begg’s rank correlation test and Egger’s linear regression test further indicated that there was no obvious publication bias among the included studies (*P*_Begg_ = 0.53, *P*_Egger_ = 0.74).

## Discussion

The overall results of the present study suggested that patients with a history of using ARB-based therapy were not at increased risk of cancer as compared to both patients with no history of ARB use and those who used ACEIs. However, when a subgroup analysis was conducted according to cancer site, it was observed that reduced incidence of lung cancer may be associated with ARB use. Furthermore, the combined results of included cohort studies suggested an association between ARB-based therapy and decreased risk of cancer. When the different types of ARB drugs were considered individually, no significant associations with risk of cancer were found, which was consistent with the results of analysis of 15 trials by the ARB Trialists Collaboration [12].

### ARB-based therapy and risk of cancer

The relationship between ARB-based therapy and risk of cancer has drawn significant attention since 2010, when a meta-analysis of RCTs demonstrated slightly increased incidence of cancer among patients using ARBs. However, a major limitation of the study was that cancer was not the primary outcome of the included trials. Several large observational studies have since investigated the potential relationship, yet the results of individual studies have been inconsistent. Recently, Azoulay L et al [19] showed that ARB use was not associated with increased risk of cancer relative to use of other therapies for hypertension (RR: 1.00; 95% CI: 0.96–1.03). However, ARBs were found to be associated with a small reduction in risk of colorectal cancer (adjusted RR: 0.88; 95% CI: 0.81–0.96). Rao et al showed that ARB-based therapy could decrease lung cancer incidence (HR: 0.74, 95%CI: 0.67–0.83) [16] and prostate cancer incidence (HR: 0.91, 95% CI: 0.84–0.99) [17]. Wang et al [18] likewise found a protective effect with incidence of cancer being 4% among patients using ARBs and 6% among ARB nonusers (HR: 0.58, 95% CI: 0.55–0.62).

The present analysis investigated whether increased risk of cancer was found to be associated with ARB use based on the combined results of such observational studies. Our results suggest that patients with a history of using ARBs did not experience significantly higher incidence of breast, prostate, or colorectal cancers. In fact, a marginally reduced risk of breast cancer was observed. Furthermore, a subgroup analysis considering lung cancer cases revealed that patients reporting any ARB use had a significantly lower risk of cancer at this site. Azoulay L et al [19] found that the use of ACEI and calcium channel blockers was associated with 13% and 19% increasing of lung cancer, respectively; however, compared to diuretics and/or beta-blockers, use of ARBs did not increase the incidence of lung cancer. In the other included studies [15, 16, 18, 21], the similar conclusion was revealed. Recent laboratory studies indicated that the overexpression of angiotensin II type 2 receptor gene induced cell death in lung adenocarcinoma cells [45] and angiotensin-converting enzyme 2 attenuated the metastasis of non-small cell lung cancer through inhibition of epithelial-mesenchymal transition [46], which indirectly supported our results of subgroup analysis focusing on lung cancer.

Besides the popular reported sites (breast, prostate, lung or colorectal), Wang KL et al [18] found that ARB users may be with the lower incidence of liver (RR: 0.51, 95%CI: [0.43, 0.60]) and stomach cancer (RR: 0.61, 95%CI: [0.48, 0.78]). However, another study performed by Chang CH [21] suggested that the use of ARBs was not significantly associated with the decreased incidence of liver (RR: 0.96, 95%CI: [0.64, 1.44]) and gastric cancer (RR: 0.65, 95%CI: [0.32, 1.34]) in diabetic patients. For other cancers, several studies revealed no significant association between the use of ARBs and haemotological (RR: 0.94, 95%CI: [0.74, 1.20]) [20], tobacco related malignancies (RR: 1.08, 95%CI: [0.97, 1.20]) [20], or pancreatic cancer (RR: 0.68, 95%CI: [0.14, 3.21]) [21]. Those sites of cancer should be also paid attention, which need more data to get stronger evidence.

### Sources of heterogeneity

Between-study heterogeneity was assessed using the criteria of *P* ≤ 0.10 and I^2^ ≥ 50% for Cochran’s *χ* ^2^-based Q test and the I-squared test, respectively. Due to the heterogeneity observed among included studies, pooled estimates were calculated by the random effects model for both the overall analysis and for several of the subgroup analyses. This model assumes that underlying true effects differ between studies. Sources of heterogeneity could include differences in participant characteristics across studies, study design factors, and variations in the metrics (RR versus HR) used to measure the outcome. For the present study, the use of the random effects model was expected to adequately address heterogeneity due to the lack of observable publication bias in our study. Additionally, through the sensitivity analysis, no study was found to significantly contribute to the heterogeneity; we also found no significant potential publication bias.

### Study strength and limitations

To the best of the author’s knowledge, this is the first meta-analysis of observational studies exploring the relationship between use of ARBs and risk of cancer. The results of the present analysis are intended to provide more robust evidence than any individual study; however, several limitations may still influence the findings. First, considerable heterogeneity was observed among the included studies. As indicated above, we used the random effects model to address the heterogeneity and conducted a sensitivity analysis to identify studies that contributed to it. Next, subgroup analyses by cancer site were only conducted for breast, prostate, colorectal, and lung cancers due to the limited evidence available for other sites. Lastly, our analysis was restricted to studies published in English or Chinese.

### Suggestion for further studies

Future studies should consider the association between ARB use and other cancer sites, including gastric cancer, and additional focus should be given to the rates of lung and breast cancers among ARB users.

## Conclusions

This meta-analysis suggested that ARB-based therapy was not associated with increased risk of cancer. In contrast, use of ARBs may be related to decreased incidence of lung cancer. This conclusion should be considered carefully and confirmed with further studies.

## Supporting Information

S1 Table PRISMA 2009 Checklist. (DOC)
